# Evidence, implementation, and challenges in mechanical ventilation

**DOI:** 10.36416/1806-3756/e20250071

**Published:** 2025-03-18

**Authors:** Rodrigo Cavallazzi

**Affiliations:** 1. Division of Pulmonary, Critical Care, and Sleep Disorders Medicine, University of Louisville School of Medicine, Louisville, KY, USA.

A lot of what we do in medicine is either not informed by data or not supported by conclusive research. In 49% of all Cochrane systematic reviews, the conclusion is that the data do not support either benefit or harm. Further studies are recommended in the majority of the reviews.^1^ In a more recent analysis of Cochrane reviews, it was found that for only 1 in 10 health care interventions is there a published high-quality primary outcome. Overall, only 5.6% of all health care interventions are deemed to be effective on the basis of the available data.^2^ Of course, one should not be dogmatic, and not every aspect of the care we provide requires a multicenter clinical trial. Overall, however, there is a knowledge gap for much of what we do in medicine. High-quality data generated by multicenter clinical trials are of paramount importance to inform clinicians. 

An intriguing phenomenon is that the limited number of health care interventions that are deemed effective are often not implemented in clinical practice. In a study conducted in 118 ICUs in Brazil, it was found that 42% of the mechanically ventilated patients did not receive a tidal volume ≤ 8 ml/kg of predicted body weight.^3^ Prone positioning was implemented in only 16.3% of patients with severe ARDS in an international study that included 459 ICUs in 50 countries.^4^ Another concept that is closely associated with implementation of effective health care interventions is that of variation in health care.^5^ Some degree of variation may not necessarily be harmful and could drive innovation. The problem is substantial variation, a lack of standardization, and the consequent failure to implement effective interventions consistently. 

Important steps to improve the implementation of effective interventions and reduce variation in health care include increasing the knowledge base of clinicians and adopting protocols. To that end, medical societies produce documents that summarize the medical literature and provide evidence-based recommendations such as guidelines and consensus statements. In this issue of the JBP, Ferreira et al.^6^ publish a joint statement on evidence-based practices in mechanical ventilation. The project is sponsored by two Brazilian medical societies: the *Sociedade Brasileira de Pneumologia e Tisiologia* and the *Associação de Medicina Intensiva Brasileira*. The document was produced by 75 authors with expertise in the field. It includes 38 topics. For each topic, there is a comment, which is a brief explanation of the theme to be addressed. This may be followed by one or more suggestions in the presence of at least one randomized trial with low risk of bias or existing statements endorsed by well-established health organizations, or one or more considerations in the absence of a high level of evidence. In this issue, JBP readers will find an article containing a detailed explanation of the methodology used in order to generate the document, as well as a useful and practical table highlighting the suggestions and/or considerations for each topic. The full document, which is freely available on the websites of the two societies, can be accessed through a link in the published article. 

The end result of the work by Ferreira et al.^6^ is a comprehensive, evidence-based guide to mechanical ventilation. There are core or essential topics, as well as topics that are quite unique and not easily found elsewhere, such as mechanical ventilation in pregnancy, dental care in mechanically ventilated patients, and respiratory support for patients under palliative care. Finally, there are chapters that address themes that could be considered trending because they reflect recent research or renewed interest. These include mechanical ventilation in patients with COVID-19, awake prone positioning, and patient self-inflicted lung injury. The results of studies by Brazilian scientists inform many chapters, including the use of protective ventilation to improve survival in ARDS,^7^ the benefit of low tidal volume that extends to patients without ARDS,^8^ the lack of benefit and potential harm with lung recruitment maneuvers in moderate to severe ARDS,^9^ the association of driving pressure and survival in ARDS,^10^ the relative effect of ventilator variables on mortality in ARDS,^11^ the effect of spontaneous breathing on the pleural pressure in different regions of the lung during mechanical ventilation,^12^ and the effect of assisted breaths on lung histology in patients ventilated with pressure-limited modes.^13^

The authors recognize that some of the suggestions and considerations might be difficult to adopt widely in Brazilian ICUs because of the lack of resources. For example, take two technologies that have been game changers in the ICU. One is video laryngoscopy, which has recently been shown to be superior to direct laryngoscopy for critically ill adults undergoing endotracheal intubation.^14^ In the document, video laryngoscopy is part of the difficult airway and failed airway algorithms. Another is high-flow nasal cannula, for which there is now a large body of evidence showing it is either noninferior^15^ or superior^16^ to other forms of oxygen delivery in acute respiratory failure. These technologies may be found in select major academic centers or large private hospitals but are unlikely to be currently found elsewhere in Brazil. A recent publication by the *Associação de Medicina Intensiva Brasileira* shows that there is an enormous regional disparity in ICU resources in Brazil. Although there are 7.35 intensivists per 100,000 population in southeastern Brazil, there are only 2.01 per 100,000 population in northern Brazil and 3.02 per 100,000 population in northeastern Brazil. Although there are 42.58 ICU beds per 100,000 population in southeastern Brazil, there are only 27.52 per 100,000 population in northern Brazil and 29.28 per 100,000 population in northeastern Brazil.^17^ Early in the COVID-19 pandemic, invasive ventilatory support outside of the ICU was provided to 13% of invasively ventilated patients with COVID-19 in southeastern Brazil; this contrasts with 17% in northern Brazil and 16% in northeastern Brazil. The ICU mortality for patients with COVID-19 was 49% in southeastern Brazil; this contrasts with a staggering 79% in northern Brazil and 66% in northeastern Brazil.^18^ Resource disparities also exist when capitals are compared with the countryside or when the public health system is compared with the private sector.^17^ How can these inequalities be factored in when an attempt is made to produce a unifying evidence-based document on mechanical ventilation? I agree with the approach of the authors, who favored the inclusion of evidence-based interventions even if they require extensive expertise or advanced technologies. As the authors point out, it is the hope that the suggestions and considerations in the document will inform health policy and ultimately improve access to ICUs that are adequately structured, equipped, and staffed. 

The document is of interest to a broad readership, including clinicians, nurses, and respiratory therapists working in the ICU. Table 2, which summarizes the suggestions and considerations, can be easily adapted into a checklist for use at bedside. I suggest that Table 2 and the full document both be added to the curriculum of the Brazilian medical residencies. Ideally, the document should be periodically updated at short intervals-and this might be a challenge. The authors and the medical societies deserve congratulations for such an important work. 
